# Functional Magnetic Neuromuscular Stimulation vs. Routine Physiotherapy in the Critically Ill for Prevention of ICU Acquired Muscle Loss: A Randomised Controlled Trial

**DOI:** 10.3390/medicina60101724

**Published:** 2024-10-21

**Authors:** Anej Skočir, Alja Jevšnik, Lidija Plaskan, Matej Podbregar

**Affiliations:** 1 Department for Medical ICU, General and Teaching Hospital Celje, 3000 Celje, Slovenia; anejskocir@gmail.com; 2 Department for Medical Rehabilitation, General and Teaching Hospital Celje, 3000 Celje, Slovenia; 3 Department for Internal Medicine, Medical Faculty, University of Ljubljana, 1000 Ljubljana, Slovenia

**Keywords:** functional magnetic stimulation, skeletal muscle, ICU acquired weakness, muscle ultrasound

## Abstract

*Background and Objectives*: Muscle loss is a known complication of ICU admission. The aim of the study was to investigate the effect of neuromuscular functional magnetic stimulation (FMS) on quadriceps muscle thickness in critically ill patients. *Materials and Methods*: Among ICU patients one quadriceps was randomized to FMS (Tesla Stym, Iskra Medical, Ljubljana, Slovenia) stimulation and the other to control care. Quadriceps thickness was measured by ultrasound (US) in transversal and longitudinal planes at enrolment, Days 3–5, and Days 9–12. The trial stopped early following an interim analysis comparing muscle thickness differences between groups using repeated measures ANOVA. *Results*: Of 18 patients randomized, 2 died before completing the trial. The final analysis reported included 16 patients (female 38%, age 68 ± 10 years, SOFA 10.8 ± 2.7). Three mild skin thermal injuries were noted initially, which were later avoided with proper positioning of FMS probe. Primary outcome comparison showed that quadriceps thickness in transversal and longitudinal planes decreased in the non-stimulated legs and, but it did not change in FMS legs (−4.1 mm (95%CI: −9.4 to −0.6) vs. −0.7 mm (95%CI: −4.1 to −0.7) (*p* = 0.03) and −4.4 mm (95%CI: −8.9 to −1.1) vs. −1.5 mm (95%CI: −2.6 to −2.2) (*p* = 0.02), respectively) (ANOVA difference between groups *p* = 0.036 and 0.01, respectively). *Conclusions:* In the critically ill, neuromuscular FMS is feasible and safe with precautions applied to avoid possible skin thermal injury. FMS decreases the loss of quadriceps muscle thickness.

## 1. Introduction

In critical illness, intensive-care-unit-acquired weakness (ICU-AW) is a known complication [1,2] with an estimated 50% rate of muscle weakness [3,4]. Patients with ICU-AW stay longer on mechanical ventilation. It causes persistent functional impairment, lasting even after hospital discharge [5,6]. The cause of ICU-AW is multifactorial form systemic inflammation, sepsis, immobilisation, sedation, hyperglycaemia, and exposure to neuromuscular blocking agents and corticosteroids [7,8], which all lead to decreases in muscle mass and strength [9]. There are different strategies to ameliorate the ICU-AW including passive mobilisation [10,11], shorter time of depth sedation and adequate analgesia for promoting faster awakening allowing active movements [12].

The ICU physiotherapy also includes peripheral transcutaneous electrical stimulation [13], something that has been found effective in non-critically ill patients [14]. Despite electrical stimulation is a method for preventing ICU-AW, it has some important limitations [13]. It is effective in critically ill patients when the stimulation starts within the first seven days of admission and is not effective in very acute conditions [15]. Electrical stimulation can induce visible muscle contractions in 75–80% of patients, probably due to tissue oedema over the muscles acting as insulation [16]. Electrical stimulation is also painful, what is difficult to assess and avoid in the critically ill [17]. Thus, an alternate approach is needed.

Transcutaneous neuromuscular functional magnetic stimulation (FMS) differs from electrical stimulation. FMS uses a magnetic applicator instead of electrodes for muscle tissue stimulation [18] and is known to be less painful [17]. An electric coil installed in the applicator generates a magnetic field that propagates into the human body, inducing electric currents [18]. These induced currents artificially propagate a signal along neurons inducing muscle contraction. Despite transcutaneous electrical stimulation and FMS have the same triggering mechanism in the nerve cell, they differ in the method of energy delivery [13]. Unlike electrical stimulation, which rarely reaches structures more than 12 mm deep, FMS penetrates deeper into the body without direct contact of the applicator with the skin. That is why FMS can be performed through clothing and bandages [19]. FMS also causes little or no pain, because it does not cause a high concentration of electric current at the point of entry into the body through the skin [16]. It was shown that FMS can prevent inactivation atrophy of skeletal muscles in the mobilised limb of rats [20]. It was also shown that in humans transcutaneous FMS of the quadriceps is a safe and painless method [21]. In patients with COPD quadriceps FMS was successfully applied and it increased size of slow-twitch muscle fibres, increased contraction force by 17% and improved quality of life [22,23]. It was previously also shown that lumbar repetitive peripheral magnetic stimulation in lumbar region fosters nerve regeneration and motor recovery in patients with lumbar radiculopathy [24].

In the above background, the primary purpose of the study was to evaluate the effect of FMS in critically ill patients to prevent quadriceps atrophy.

## 2. Materials and Methods

### 2.1. Study Design

The current study was a single-centre, parallel, two-arm, open-label, randomised controlled trial. It was conducted in an 11-bed, level 3, medical ICU at the General and Teaching Hospital Celje, Slovenia during a 6-month period (from December 2023 to April 2024). The study was approved by the Institutional Review Board of General Hospital Celje (No. 61/2023/3, 25 October 2023). It was prospectively registered (NCT06368908, 16 April 2024). Informed consent was signed by relative carers before inclusion and confirmed by the patient after gaining conciseness. The trial is reported according to CONSORT guidelines [25].

### 2.2. Participants

Critically ill patients after 2 to 3 days of ICU admission, whose treatment was expected to require at least 10 days in the ICU, were included in the study when the muscle ultrasound team (A.S., M.P.) was available. Exclusion criteria were as follows: age < 18 years of age, implanted electrical devices affected by magnetic fields, expected ICU survival of less than 5 days, pregnancy, bone and tissue injuries of legs where standard physiotherapy cannot be performed, high-dose corticosteroid application (equivalent to >300 mg hydrocortisone per day), continuous or intermittent muscle relaxants use, obesity (BMI over 40 kg/m^2^) or cachexia (BMI less than 20 kg/m^2^ or loss of 5% body weight over 12 months), brain death, and inability to obtain informed consent.

### 2.3. Randomisation and Baseline Data

After the recruitment of the patient, the right or the left leg was randomised to transcutaneous FMS while the other leg was allocated to control care. Research Randomizer (Research Randomizer, www.randomizer.org (accessed on 15 September 2024)) was used to create the allocation sequence. Allocation data for each consecutive patient was saved in separate closed envelope. Trial investigators enrolling the participants were kept blind to the random allocation until after the consent was obtained. ICU and 28-day basic demographic data, ethology, and severity of disease outcome data were collected from the hospital electronic database BIRPIS21 (SRCInfonet, Kranj, Slovenia). Laboratory analysis on admission was carried out in the laboratory of our institution. The worst of the first 24 h SOFA (sepsis-related organ failure assessment) [26] and APACHE II (acute physiology and chronic health evaluation) [27] score were used.

### 2.4. Transcutaneous Neuromuscular Functional Magnetic Stimulation

Skeletal muscle FMS was performed above randomised quadriceps using a magnetic stimulator (Tesla stym, Iskra Medical, Ljubljana, Slovenia). For FMS, a predefined ICU program was used (pulse trains (30 Hz) with power ranging from 0.5 to 2.5 (100%) T, 6 s long stimulation trains, and a duty cycle varying from 1:1 to 1:10). Muscles were stimulated with a magnetic field intensity that triggers touchable and visible contraction. For touchable detection of muscle contraction, the investigator’s hand was put on the skin over the contracting muscle close to stimulation probe. The stimulation was performed for 55 min per randomised selected limb 5 days per week. The percent of maximal power of magnetic stimulation used for each stimulation and the number of stimulations per patient were recorded. FMS (frequency: 30 Hz, power: 1.6 T) can generate approximately 72 ± 5% of the maximal spontaneous contraction force of the quadriceps [22].

Temperatures of the working head of magnetic stimulator, connecting cables, skin under working head of stimulator at the end of stimulation cycle and temperature of contralateral not stimulated skin were measured with IR camera (GTC 600C, Bosch, Gerlingen, Germany) in one patient after AE detection.

### 2.5. Routine Treatment and Physiotherapy

Treatment data (i.e., maximal noradrenaline dose, duration of mechanical ventilation) was collected from the intensive care information system (Centricity Critical Care, GE healthcare, Chicago, IL, USA). The feeding of the patients according to European Society for Clinical Nutrition and Metabolism (ESPEN) guidelines was at discretion of the treating physicians [28]. The physiotherapy of the patients according to current recommendations was at the discretion of the ward physiotherapists [29]. Twice per day, classical physiotherapy was performed (passive stretching), followed by active bed exercising with both legs, sitting, and early mobilisation as soon as possible.

### 2.6. Quadriceps Muscles Thickness Measurement by Ultrasound and Strength Testing

Measurements of quadriceps thickness and cutaneous/subcutaneous tissues were taken three times on each leg (first: upon enrolment in the study; second: between Days 3–5 after enrolment; and third: between Days 9–12 after enrolment) at the measuring point, which was set as follows. The patient was in the supine position, leg in a neutral position, knee extended with fingers pointing up. On the thighs, the transversal measurement line was marked circumferential at the lower 1/3 of the distance between the spino-iliac point and the medal of the patella. The longitudinal measurement line was set, where the rectus femoris, vastus intermedius and femur were centrally located in the transversal plane. All further measurements we performed at the crossing of transversal and longitudinal measuring line, marked with a permanent marker [30]. Ultrasound (US) (Vivid 70, GE Healthcare, Chicago, IL, USA) examination was performed with a linear probe of 8–12 MHz; ultrasound settings (frequency 12 Hz, gain: 55 dB, dynamic range: 75) were kept constant for all patients, with depth adjusted only in the case of larger muscle thickness. Measurements were performed in transverse and longitudinal muscle sections with minimal compression of the US probe, the probe was perpendicular to get the shortest distance to avoid oblique scanning. Quadriceps thickness (both rectus femoris and vastus intermedius together) was defined as the distance between the superior border of the muscle and the cortex of the femur. US measurements were performed by A.S. and M.P, who are educated to perform US in different body regions of critically ill including muscles. To avoid subjectivity in measurement, the videos and measurements were reevaluated by both operators together on EchoPack ultrasound analysis software v206 (GE Healthcare, Chicago, IL, USA) to reach the consent on the final measurement.

### 2.7. Outcomes and Monitoring of Side Effects and Adverse Effects

Primary outcome was the effectiveness of the FMS on quadriceps thickness in critically ill patients. Secondary outcome was the muscle strength between FMS stimulated and not-stimulated leg. At the end of the study, in patients who were able to cooperate (Ramsay Sedation Scale: 2 or 3 points) [31], the muscle strength of both legs was tested according to the Medical Research Council (MRC) [32]. The test was performed by a trained physiotherapist (A.J.). ECG monitoring (CarescapeB850, GE Healthcare, USA) was observed to detect interactions between FMS and monitoring signals. Possible local skin changes as redness of the skin and local burns under FMS probe were visually detected and recorded. Skin redness and blisters < 2 cm, up to superficial second degree burns, were defined as mild adverse effects (AE) according to burs classification [33]. Severe adverse effects (SAE) were all higher-grade thermal injuries of the skin and subcutaneous tissue compared to mild AE. The AE were independently individually reviewed and treated by two team-nurses, who had additional education of wound management (post-registration qualification of wound management—European qualification framework level 6, Slovenian Wounds Management Society, dors.si) and two intensivists with more than 5 years ICU working experience including also surgical ICU.

### 2.8. Sample Size Estimation and Statistical Analysis

The indicated sample size in the trial registry was 20. The sample size was estimated on previous data [29], assuming the mean difference of muscle thickness between the FMS and control groups at the end of the study of 5 mm with standard deviation of 5 mm. At this level of effect, 17 patients would have been required in the study with type I error level of 0.05 (*p*-value threshold 0.05) and a type II error of 0.20 (power 80%), we added 3 patients due to potential loss of patients after recruitment. There were mild thermal skin injuries noted initially in the 3 (2nd, 4th, and 5th) of the first 5 patients recruited. Thus, it was decided that interim analyses after additional inclusion of 6 patients should be carried out to maximise safety. The trial was stopped earlier than planned, due to the interim analysis showing statistically significant effects on quadriceps thickness.

Normality of data distribution was tested by the D’Agostino–Pearson test. Normally distributed data are presented as mean (±standard deviation) for metric variables, absolute and relative frequencies for categorical variables. Not-normally distributed data is presented as median (95%CI). The Mann–Whitney test for independent samples or the Wilcoxon test for paired samples were used to compare two groups. The effect of treatment compared to control on repeated measurements of quadriceps thickness during the study was analysed by repeated measures ANOVA. MedCalc ver. 12.5 (MedCalc Software Ltd., Ostend, Belgium) was used for sample size estimation and statistical analysis. A *p*-value < 0.05 was considered to define statistical significance.

## 3. Results

**Figure 1:**
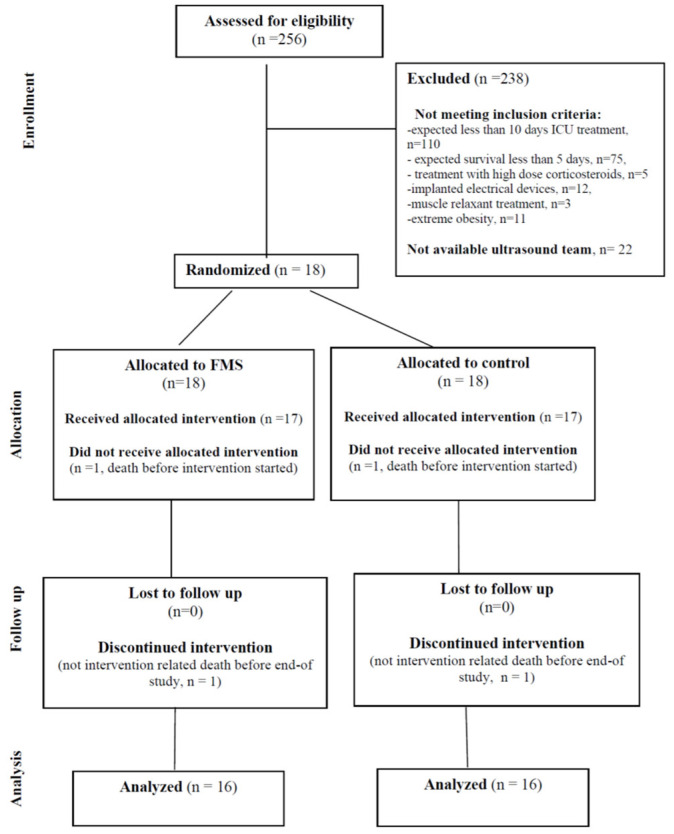
The CONSORT diagram showing the flow of participants through each stage of a randomised trial.

There were 18 patients randomised, of whom 2 patients died before completing the study. The flow of participants through each stage of a randomized trial is presented in Figure 1. The primary outcome data were available for 16 patients.

Demographic data, severity of disease, treatment and outcome of patients is presented in Table 1. In all patients FMS induced palpable quadriceps contractions, confirming feasibility of FMS in critically ill patients. Initial power of FMS was 100% (95%CI; 94–100). During study period 10 (95%CI: 9–10) FMS were performed in each patient. Median cutaneous/subcutaneous tissue was not different between FMS compared to non-stimulated leg (12.8 mm (95%CI: 5.0–15.8) vs. 11.9 mm (95%CI: 6.2–16.1), *p* = 0.6).

**Figure 2:**
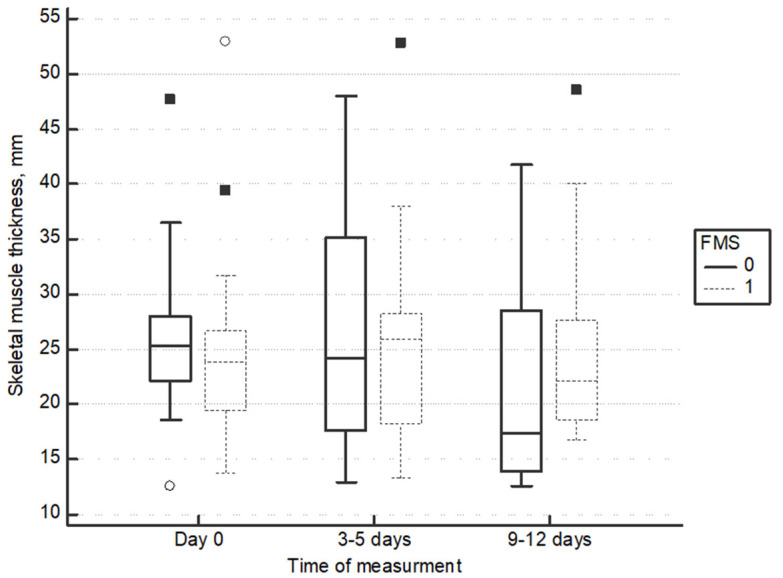
Change of quadriceps thickness in transversal plane.

Quadriceps thickness in transversal plane decreased in the non-stimulated legs, but it did not change in FMS legs (−4.1 mm (95%CI: −9.4 to −0.6) vs. −0.7 mm (95%CI: −4.1 to −0.7) (*p* = 0.03)) (repeated measurements ANOVA difference between groups F = 3.52, *p* = 0.036) (Figure 2).

**Figure 3:**
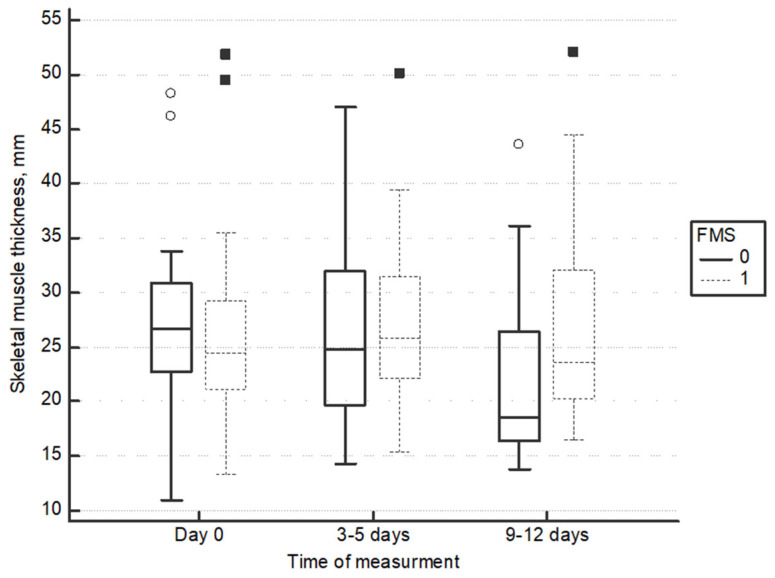
Change of quadriceps thickness in longitudinal plane.

Quadriceps thickness in longitudinal plane decreased in the non-stimulated legs, but it did not change in FMS legs (−4.4 mm (95%CI: −8.9 to −1.1) vs. −1.5 mm (95%CI: −2.6 to −2.2) (*p* = 0.02)) (repeated measurements ANOVA difference between groups F = 5.06, *p*= 0.01) (Figure 3). Details of quadriceps thickness measurements is available in Table 2.

At the end of the study, in 8 cooperative patients (Ramsay Sedation Scale: 2 or 3 points), leg muscle strength was tested (Table 3). Hip flexion, knee extension, and ankle dorsiflexion strength were higher on FMS-stimulated legs.

Concerning adverse reactions/events, during FMS there were stimulation spikes detected on the ECG signal on the bed-side monitoring system, but there was no triggering of any alarms. All other signals (i.e., invasive arterial pressure, frequency of respiration, arterial haemoglobin oxygen saturation) were undisturbed. The stimulation probe and connecting cable are heated due to high current induction, the probe is cooled by specially designed air channels and ventilator on the top of the probe (detained thermal analysis of probe/connecting cable/skin after stimulation with IR camera is available in the Supplementary Materials). At the beginning of study, in the first 5 patients, the FMS stimulation head was put above the thigh, which was covered by a towel. There were mild thermal skin injuries noted initially in the 3 (2nd, 4th, and 5th) of the first 5 patients recruited. In the 2nd recruited patient, we detected redness of the skin directly under the stimulation head, which disappeared by local cooling. In the 5th patient, the probe was accidentally covered with the sheet by nursing staff, the redness of skin and blister < 2 cm was detected. The blister was left alone, and the area was cooled and covered with hydrocolloid dressing (Granuflex, ConvaTec, Bridgewater, NJ, USA). The side-effect was classified as mild AE. FMS was continued the next day on a slightly different spot so that the thermal injury was not under the stimulation head. In the 4th patient, a blister < 2 cm on the sole of the foot was found, it has been retrospectively connected with the injury due to connection cable between probe and stimulator. The treatment was conservative with hydrocolloid dressing (Granuflex, ConvaTec, USA) and it was classified as mild AE. In the continuum of the study, the position of the stimulation head and connecting cable during stimulation were changed; the head was put on specially designed holder, a few millimetres above the towel covering the quadriceps, the connecting cable was not in contact with the patient anymore (Figures S1 and S2). No further thermal injuries were detected to the end of the study.

## 4. Discussion

The current study confirmed that neuromuscular FMS in critically ill patients is feasible and safe with precautions used to avoid thermal skin injuries. FMS decreases the loss of quadriceps thickness. There was also a difference in leg muscle strength as a secondary outcome in favour of FMS, but the muscle strength testing was performed only in 8 cooperative patients. Transcutaneous skeletal muscle FMS could become an additional therapeutic modality to ameliorate quadriceps atrophy influencing the short- and long-term outcomes of the critically ill.

We registered the trial prospectively and reported according to CONSORT guidelines [25]. Our study stopped early, but this was due to the demonstration of a statistically significant difference in effectiveness, not due to safety concerns. The interim analysis was not originally planned in the registered protocol. However, in any trial involving human subjects, safety is the primary ethical concern. As terminal injury had been initially observed, interim analyses were introduced for monitoring. The changes made to the intervention protocol eliminated the thermal injury concern. However, as the interim analysis showed a significant effect on muscle thickness, the registered primary outcome, the trial was stopped early. Muscle thickness was registered as the primary outcome because a recent study had confirmed that every 1% loss of quadriceps thickness over the first week of critical illness is associated with 5% higher odds of 60-day mortality [34]. In rehabilitation, neuromuscular FMS serves to counteract muscle atrophy and to support relearning of movement sequences; orthodromic 4 signals travelling from the periphery back to the central nervous system seem to trigger supportive neuroplastic effects [35]. Given the relationship of muscle atrophy with mortality, it was not ethically justifiable to continue the trial as the control group would have experienced a worse outcome compared to intervention in the remaining sample size after the interim analysis had demonstrated effectiveness for the primary outcome.

The major advantage of skeletal muscle FMS compared to electrical stimulation is that it is known to be less painful [16,36]. In our study, stimulation FMS protocol used a similar protocol as electrical stimulation in a previous study and was already predefined in the stimulation apparatus [15,16,36,37]. Significantly greater muscle strength and shorter weaning from mechanical ventilation was reported in patients who received electrical stimulation in addition to standard rehabilitation treatment in comparison to patients who receive only standard rehabilitation treatment [15]. Electrical stimulation alters muscle fibres proportions; changes depend on the selected stimulation parameters, stimulation duration, and muscle innervation. This decreases the proportion of fast, glycolytic fibres (type II), which are seen dominantly in less active individuals, and increases the proportion of more endurance-oriented, slow-contracting muscle fibres (type I) [15]. Similar changes in skeletal muscle architecture were detected in critically ill patients during ICU stay [38]. The effect of FMS on different types of muscle fibres in critically ill patients is currently unknown.

In our trial, leg muscle strength at the end of the study in a sub-group of cooperative patients was different between FMS and not-stimulated legs, in favour of FMS. The difference in quadriceps thickness can implicate the possibility of increased strength as was shown in a recent report [39]. Measuring maximal muscle strength with magnetic stimulation is currently not validated for the critically ill [40].

There are different approaches to assessing the skeletal muscles using ultrasound in the critically ill [41]. In the current study, quadriceps thickness was measured; we measured quadriceps rectus femoris and vastus intermedius together. Fivez T et al., using the same methodology as in our study, confirmed ultrasound as a reliable tool for early detection of muscle mass wasting in critically ill adults (median absolute inter-observer variability 0.5 mm (IQR: 0.3–0.9), absolute intra-observer accuracy 95%CI: 2.2 mm) [30]. Other studies confirmed excellent intra-observer and inter-observer reliability of quadriceps thickness measurements [42,43,44].

In our study, the muscle thickness measurements were done while applying minimal compression of the US probe to avoid possible measurement errors [41]. Compression, especially maximal, can alter the appearance of myofascial structures and has been shown to compromise the validity of US muscle parameters [45,46]. The trend of quadriceps thickness change during our study on the non-stimulated leg are in limits as previously observed [47]. Special care was taken so that US skeletal muscle measurements were performed while the patient was supine with knee in extension and toes pointing to the ceiling to ensure that measurements were taken at the same muscle location in subsequent evaluations [45]. Dual energy X-ray absorptiometry, computer tomography scanning, and magnetic resonance imaging could be used in further skeletal muscle FMS studies to independently confirm our observations, preservation of muscle volume/thickness.

Heat is produced during magnetic field generation in a magnetic stimulator, which was used in our study. The excessive heat is removed by a special design. Despite this, the stimulation head temperature at the end of stimulation reached around 40 °C (Figures S3–S5 in Supplementary Materials). This high temperature increases the skin temperature under the stimulation head. This is especially important in fragile, not-moving, critically ill patients without verbal contact, where the skin thermal injury can be induced, as was the case in the beginning of the current study. Before starting the stimulation, the stimulation head should not lay directly on the skin; we placed a towel on the skin first; a few millimetres above the towel, there was a stimulation head fixed by a specially designed holder. There should not be any contact between the patient and the stimulation head or connecting cable to avoid skin thermal injury.

The pathophysiology of ICU-AW is complex, including not only muscle changes but also to long-term neural impairment [48]. Lumbar repeated peripheral magnetic stimulation improves nerve regeneration in lumbar radiculopathy [24]. Further research should explore the effects of combined therapy of FMS and lumbar repeated peripheral magnetic stimulation to enhance corticospinal excited motor recovery during or after ICU care. This combination could offer a more comprehensive approach to patient rehabilitation.

Further independent studies need to confirm our results and to find optimal stimu2lation protocols of FMS (i.e., duration of stimulation, frequency of stimulation) for the critically ill. In the future, the effect of FMS should be tested on different skeletal muscle groups and different magnetic coil designs should be applied [49].

## 5. Conclusions

Skeletal muscle FMS is feasible in the critically ill. Precautions should be applied to avoid possible skin thermal injury. FMS decreases the loss of quadriceps thickness. FMS could be a promising additional method to classical physiotherapy, electrical stimulation, and early mobilisation for ameliorating muscle atrophy in the critically ill.

## Figures and Tables

**Table 1:** Demographic data, severity of disease, treatment and outcome.

	Values (n = 16)
Gender (female/male), n (%)	6/10 (37/63)
Age, years	68 ± 10
BMI, kg/m^2^	31.5 ± 6.3
WHO performance at home, points	1.0 ± 0.9
**Severity of disease and treatment**	
Septic shock/sepsis, n (%)	10/6 (63/37)
Acute respiratory failure, n (%)	15 (94)
Acute renal failure, n (%)	14 (88)
Acute circulatory failure, n (%)	7 (44)
Acute liver failure, n (%)	3 (19)
SOFA score at admission, points	10.8 ± 2.7
APACHE II at admission, points	26.0 ± 7.2
Lactate at admission	2.2 [95%CI: 1.7–3.2]
Maximal noradrenalin dose, µg/kg/min	0.41 ± 0.21
Haemodialysis support, n (%)	4 (25%)
Duration of mechanical ventilation, days	8.2 ± 5.6
Duration of ICU stay, days	12.7 ± 6.2
**Outcome**	
ICU survival, n (%)	15 (94)
28-day survival, n (%)	8 (50)

APACHE II—acute physiology and chronic health evaluation II, BMI—body mass index, ICU—intensive care unit, WHO—world health organization. SOFA—sepsis-related organ failure assessment.

**Table 2:** Quadriceps muscle thickness.

		Day of	Measurement			Statistics	
Ultrasound Plane		Day 0	Day 3–5	Day 9–12	Day 0 vs. Day 3–5 *p*-Value	Day 0 vs. Day 9–12 *p*-Value	Day 3–5 vs. Day 9–12 *p*-Value
Transversal plane	No-FMS	25.3 [22.4–27.8]	24.3 [17.7–35.1]	17.4 [13.7–27.7]	0.78	0.003 *	0.001 *
	FMS	23.9 [19.5–26.6]	25.9 [18.5–28.3]	22.2 [18.8–27.5]	0.5	0.6	0.5
Longitudinal plane	No-FMS	26.6 [23.0–30.4]	24.9 [19.8–31.8]	18.5 [16.4–26.2]	0.6	0.002 *	0.002 *
	FMS	24.5 [21.3–29.2]	25.9 [22.4–31.1]	23.7 [20.4–31.6]	0.9	0.6	0.9

Data are presented in millimetres as median (95CI: interval), n = 16, * statistically significant difference *p* < 0.05.

**Table 3:** Muscle strength assessment according to the Medical Research Scale.

	No FMS (n = 8)	FMS (n = 8)	Statistics *p*
Hip flexion	3.0 (95%CI: 1.0–3.4)	3.5 (95%CI: 2.0–5.0)	0.03 *
Knee extension	3.0 (95%CI: 1.8–4.0)	4.0 (95%CI: 3.0–5.0)	0.02 *
Ankle dorsiflexion	3.0 (95%CI: 1.8–3.2)	4.0 (95%CI: 2.8–5.0)	0.02 *

FMS—functional magnetic stimulation, * statistically significant difference *p* < 0.05.

## Data Availability

The data that support the findings of this study are not openly available due to the reason of patient privacy and are available from the corresponding author upon request.

## Supplementary Materials

The following supporting information can be downloaded at: https://www.mdpi.com/article/10.3390/medicina60101724/s1, CONSORT 2010 checklist of information to include when reporting a randomised trial; Figure S1. Application of stimulation head, before special holder for stimulation head was applied; Figure S2. Application of stimulation head after use of special holder; Figure S3. Thermal imaging of stimulation head immediately after FMS; Figure S4. Thermal imaging of stimulation head holder during FMS, Figure S5. Thermal imaging of connection cable during FMS.

## References

[1] De Jonghe B., Sharshar T., Lefaucheur J.P., Authier F.J., Durand-Zaleski I., Boussarsar M., Cerf C., Renaud E., Mesrati F., Carlet J. (2002). Paresis Acquired in the Intensive Care Unit: A Prospective Multicenter Study. JAMA.

[2] Puthucheary Z.A., Rawal J., McPhail M., Connolly B., Ratnayake G., Chan P., Hopkinson N.S., Phadke R., Dew T., Sidhu P.S. (2013). Acute Skeletal Muscle Wasting in Critical Illness. JAMA.

[3] Jaber S., Jung B., Matecki S., Petrof B.J. (2011). Clinical Review: Ventilator-Induced Diaphragmatic Dysfunction—Human Studies Confirm Animal Model Findings!. Crit. Care.

[4] Tobin M.J., Laghi F., Jubran A. (2010). Narrative Review: Ventilator-Induced Respiratory Muscle Weakness. Ann. Intern. Med..

[5] Herridge M.S., Cheung A.M., Tansey C.M., Matte-Martyn A., Diaz-Granados N., Al-Saidi F., Cooper A.B., Guest C.B., Mazer C.D., Mehta S. (2003). One-Year Outcomes in Survivors of the Acute Respiratory Distress Syndrome. N. Engl. J. Med..

[6] Cheung A.M., Tansey C.M., Tomlinson G., Diaz-Granados N., Matte A., Barr A., Mehta S., Mazer C.D., Guest C.B., Stewart T.E. (2006). Two-Year Outcomes, Health Care Use, and Costs of Survivors of Acute Respiratory Distress Syndrome. Am. J. Respir. Crit. Care Med..

[7] Winkelman C. (2010). the Role of Inflammation in ICU-Acquired Weakness. Crit. Care.

[8] Weber-Carstens S., Deja M., Koch S., Spranger J., Bubser F., Wernecke K.D., Spies C.D., Spuler S., Keh D. (2010). Risk Factors in Critical Illness Myopathy During the Early Course of Critical Illness: A Prospective Observational Study. Crit. Care.

[9] Callahan L.A., Supinski G.S. (2009). Hyperglycemia and Acquired Weakness in Critically Ill Patients: Potential Mechanisms. Crit. Care.

[10] Wiles L., Stiller K. (2010). Passive Limb Movements for Patients in an Intensive Care Unit: A Survey of Physiotherapy Practice in Australia. J. Crit. Care.

[11] Fan E. (2012). Critical Illness Neuromyopathy and the Role of Physical Therapy and Rehabilitation in Critically Ill Patients. Respir. Care.

[12] Juern J.S. (2012). Removing the Critically Ill Patient from Mechanical Ventilation. Surg. Clin. N. Am..

[13] Parry S.M., Berney S., Granger C.L., Koopman R., El-Ansary D., Denehy L. (2013). Electrical Muscle Stimulation in the Intensive Care Setting: A Systematic Review. Crit. Care Med..

[14] Bax L., Staes F., Verhagen A. (2005). Does Neuromuscular Electrical Stimulation Strengthen the Quadriceps Femoris? A Systematic Review of Randomised Controlled Trials. Sports Med..

[15] Gerovasili V., Stefanidis K., Vitzilaios K., Karatzanos E., Politis P., Koroneos A., Chatzimichail A., Routsi C., Roussos C., Nanas S. (2009). Electrical Muscle Stimulation Preserves the Muscle Mass of Critically Ill Patients: A Randomized Study. Crit. Care.

[16] Rodriguez P.O., Setten M., Maskin L.P., Bonelli I., Vidomlansky S.R., Attie S., Frosiani S.L., Kozima S., Valentini R. (2012). Muscle Weakness in Septic Patients Requiring Mechanical Ventilation: Protective Effect of Transcutaneous Neuromuscular Electrical Stimulation. J. Crit. Care.

[17] Han T.R., Shin H.I., Kim I.S. (2006). Magnetic Stimulation of the Quadriceps Femoris Muscle: Comparison of Pain with Electrical Stimulation. Am. J. Phys. Med. Rehabil..

[18] Goetz S.M., Truong C.N., Gerhofer M.G., Peterchev A.V., Herzog H.G., Weyh T. (2013). Analysis and Optimization of Pulse Dynamics for Magnetic Stimulation. PLoS ONE.

[19] Evans B.A., Litchy W.J., Daube J.R. (1988). The Utility of Magnetic Stimulation for Routine Peripheral Nerve Conduction Studies. Muscle Nerve.

[20] Shimada Y., Sakuraba T., Matsunaga T., Misawa A., Kawatani M., Itoi E. (2006). Effects of Therapeutic Magnetic Stimulation on Acute Muscle Atrophy in Rats After Hindlimb Suspension. Biomed. Res..

[21] Kremenic I.J., Ben-Avi S.S., Leonhardt D., McHugh M.P. (2004). Transcutaneous Magnetic Stimulation of the Quadriceps via the Femoral Nerve. Muscle Nerve.

[22] Bustamante V., Lopez de Santa Maria E., Gorostiza A., Jimenez U., Galdiz J.B. (2010). Muscle Training with Repetitive Magnetic Stimulation of the Quadriceps in Severe Copd Patients. Respir. Med..

[23] Bustamante V., Casanova J., Lopez de Santamaria E., Mas S., Sellares J., Gea J., Galdiz J.B., Barreiro E. (2008). Redox Balance Following Magnetic Stimulation Training in the Quadriceps of Patients with Severe COPD. Free Radic. Res..

[24] Savulescu S.E., Berteanu M., Filipescu I., Beiu C., Mihai M.M., Popa L.G., Popescu S.I., Balescu I., Bacalbasa N., Popescu M.N. (2021). Repetitive Peripheral Magnetic Stimulation (Rpms) in Subjects with Lumbar Radiculopathy: An Electromyography-guided Prospective, Randomized Study. In Vivo.

[25] Butcher N.J., Monsour A., Mew E.J., Chan A.W., Moher D., Mayo-Wilson E., Terwee C.B., Chee A.T.A., Baba A., Gavin F. (2022). Guidelines for Reporting Outcomes in Trial Reports: The Consort-Outcomes 2022 Extension. JAMA.

[26] Vincent J.L., Moreno R., Takala J., Willatts S., De Mendonca A., Bruining H., Reinhart C.K., Suter P.M., Thijs L.G. (1996). The Sofa (Sepsis-Related Organ Failure Assessment) Score to Describe Organ Dysfunction/Failure. On behalf of the Working Group on Sepsis-Related Problems of the European Society of Intensive Care Medicine. Intensive Care Med..

[27] Knaus W.A., Draper E.A., Wagner D.P., Zimmerman J.E. (1985). APACHE II: A Severity of Disease Classification System. Crit. Care Med..

[28] Singer P., Blaser A.R., Berger M.M., Calder P.C., Casaer M., Hiesmayr M., Mayer K., Montejo-Gonzalez J.C., Pichard C., Preiser J.C. (2023). ESPEN Practical and Partially Revised Guideline: Clinical Nutrition in the Intensive Care Unit. Clin. Nutr..

[29] Sommers J., Engelbert R.H., Dettling-Ihnenfeldt D., Gosselink R., Spronk P.E., Nollet F., van der Schaaf M. (2015). Physiotherapy in the Intensive Care Unit: An Evidence-Based, Expert Driven, Practical Statement and Rehabilitation Recommendations. Clin. Rehabil..

[30] Fivez T., Hendrickx A., Van Herpe T., Vlasselaers D., Desmet L., Van den Berghe G., Mesotten D. (2016). an Analysis of Reliability and Accuracy of Muscle Thickness Ultrasonography in Critically Ill Children and Adults. JPEN J. Parenter Enteral. Nutr..

[31] Ramsay M.A., Savege T.M., Simpson B.R., Goodwin R. (1974). Controlled Sedation with Alphaxalone-Alphadolone. Br. Med. J..

[32] Florence J.M., Pandya S., King W.M., Robison J.D., Baty J., Miller J.P., Schierbecker J., Signore L.C. (1992). Intrarater Reliability of Manual Muscle Test (Medical Research Council scale) Grades in Duchenne’s Muscular Dystrophy. Phys. Ther..

[33] Milner S.M. (2024). Classification of Burn Depth. Eplasty.

[34] Lee Z.Y., Ong S.P., Ng C.C., Yap C.S.L., Engkasan J.P., Barakatun-Nisak M.Y., Heyland D.K., Hasan M.S. (2021). Association between Ultrasound Quadriceps Muscle Status with Premorbid Functional Status and 60-Day Mortality in Mechanically Ventilated Critically Ill Patient: A Single-Center Prospective Observational Study. Clin. Nutr..

[35] Lundborg G. (2003). Nerve Injury and Repair—A Challenge to the Plastic Brain. J. Peripher. Nerv. Syst..

[36] Routsi C., Gerovasili V., Vasileiadis I., Karatzanos E., Pitsolis T., Tripodaki E., Markaki V., Zervakis D., Nanas S. (2010). Electrical Muscle Stimulation Prevents Critical Illness Polyneuromyopathy: A Randomized Parallel Intervention Trial. Crit. Care.

[37] Gondin J., Brocca L., Bellinzona E., D’Antona G., Maffiuletti N.A., Miotti D., Pellegrino M.A., Bottinelli R. (2011). Neuromuscular Electrical Stimulation Training Induces Atypical Adaptations of the Human Skeletal Muscle Phenotype: A Functional and Proteomic Analysis. J. Appl. Physiol..

[38] Wollersheim T., Woehlecke J., Krebs M., Hamati J., Lodka D., Luther-Schroeder A., Langhans C., Haas K., Radtke T., Kleber C. (2014). Dynamics of Myosin Degradation in Intensive Care Unit-Acquired Weakness During Severe Critical Illness. Intensive Care Med..

[39] Li X., Li J., Qing L., Wang H., Ma H., Huang P. (2023). Effect of Quadriceps Training At Different Levels of Blood Flow Restriction on Quadriceps Strength and Thickness in the Mid-Term Postoperative Period After Anterior Cruciate Ligament Reconstruction: A Randomized Controlled External Pilot Study. BMC Musculoskelet. Disord..

[40] O’Brien T.D., Reeves N.D., Baltzopoulos V., Jones D.A., Maganaris C.N. (2008). Assessment of Voluntary Muscle Activation Using Magnetic Stimulation. Eur. J. Appl. Physiol..

[41] Lima J., Foletto E., Cardoso R.C.B., Garbelotto C., Frenzel A.P., Carneiro J.U., Carpes L.S., Barbosa-Silva T.G., Gonzalez M.C., Silva F.M. (2024). Ultrasound for Measurement of Skeletal Muscle Mass Quantity and Muscle Composition/Architecture in Critically Ill Patients: A Scoping Review on Studies’ Aims, Methods, and Findings. Clin. Nutr..

[42] Barbosa F.D.S., Dos Santos J.L., Alves M.E.D., Alves J.A.B., Cerqueira T.C.F., De Santana Filho V.J. (2023). Inter-Examiner and Intra-Examiner Reliability of Quantitative and Qualitative Ultrasonography Assessment of Peripheral and Respiratory Muscles in Critically Ill Patients. Int. J. Environ. Res. Public Health.

[43] Sabatino A., Kooman J.P., Di Motta T., Cantarelli C., Gregorini M., Bianchi S., Regolisti G., Fiaccadori E. (2022). Quadriceps Muscle Thickness Assessed By Ultrasound Is Independently Associated with Mortality in Hemodialysis Patients. Eur. J. Clin. Nutr..

[44] Hadda V., Khilnani G.C., Kumar R., Dhunguna A., Mittal S., Khan M.A., Madan K., Mohan A., Guleria R. (2017). Intra- and Inter-observer Reliability of Quadriceps Muscle Thickness Measured with Bedside Ultrasonography by Critical Care Physicians. Indian J. Crit. Care Med..

[45] Paris M.T., Mourtzakis M., Day A., Leung R., Watharkar S., Kozar R., Earthman C., Kuchnia A., Dhaliwal R., Moisey L. (2017). Validation of Bedside Ultrasound of Muscle Layer Thickness of the Quadriceps in the Critically Ill Patient (Validum Study). JPEN J. Parenter. Enter. Nutr..

[46] Formenti P., Umbrello M., Coppola S., Froio S., Chiumello D. (2019). Clinical Review: Peripheral Muscular Ultrasound in the ICU. Ann. Intensive Care.

[47] Parry S.M., El-Ansary D., Cartwright M.S., Sarwal A., Berney S., Koopman R., Annoni R., Puthucheary Z., Gordon I.R., Morris P.E. (2015). Ultrasonography in the Intensive Care Setting Can Be Used to Detect Changes in the Quality and Quantity of Muscle and Is Related to Muscle Strength and Function. J. Crit. Care.

[48] Vanhorebeek I., Latronico N., Van den Berghe G. (2020). ICU-Acquired Weakness. Intensive Care Med..

[49] Goetz S., Kammermann J., Helling F., Weyh T., Li Z. (2022). Prediction of Force Recruitment of Neuromuscular Magnetic Stimulation From 3D Field Model of the Thigh. IEEE Trans. Neural Syst. Rehabil. Eng..

